# Polysaccharide Hydrogels Support the Long-Term Viability of Encapsulated Human Mesenchymal Stem Cells and Their Ability to Secrete Immunomodulatory Factors

**DOI:** 10.1155/2017/9303598

**Published:** 2017-10-11

**Authors:** Fahd Hached, Claire Vinatier, Pierre-Gabriel Pinta, Philippe Hulin, Catherine Le Visage, Pierre Weiss, Jérôme Guicheux, Aurélie Billon-Chabaud, Gaël Grimandi

**Affiliations:** ^1^INSERM, UMR 1229, Regenerative Medicine and Skeleton (RMeS), Université de Nantes, ONIRIS, 44042 Nantes, France; ^2^UFR Sciences Biologiques et Pharmaceutiques, Université de Nantes, 44035 Nantes, France; ^3^UFR Odontologie, Université de Nantes, 44042 Nantes, France; ^4^CHU Nantes, Pharmacie Centrale, PHU 11, 44093 Nantes, France; ^5^INSERM, UMS 016, CNRS 3556, Structure Fédérative de Recherche François Bonamy, Micropicell Facility, CHU Nantes, Université de Nantes, 44042 Nantes, France; ^6^CHU Nantes, PHU 4 OTONN, 44093 Nantes, France

## Abstract

While therapeutically interesting, the injection of MSCs suffers major limitations including cell death upon injection and a massive leakage outside the injection site. We proposed to entrap MSCs within spherical particles derived from alginate, as a control, or from silanized hydroxypropyl methylcellulose (Si-HPMC). We developed water in an oil dispersion method to produce small Si-HPMC particles with an average size of about 68 *μ*m. We evidenced a faster diffusion of fluorescein isothiocyanate-dextran in Si-HPMC particles than in alginate ones. Human adipose-derived MSCs (hASC) were encapsulated either in alginate or in Si-HPMC, and the cellularized particles were cultured for up to 1 month. Both alginate and Si-HPMC particles supported cell survival, and the average number of encapsulated hASC per alginate and Si-HPMC particle (7102 and 5100, resp.) did not significantly change. The stimulation of encapsulated hASC with proinflammatory cytokines resulted in the production of IDO, PGE_2_, and HGF whose concentration was always higher when cells were encapsulated in Si-HPMC particles than in alginate ones. We have demonstrated that Si-HPMC and alginate particles support hASC viability and the maintenance of their ability to secrete therapeutic factors.

## 1. Introduction

Mesenchymal stem cells (MSCs) have generated significant medical consideration for tissue engineering since they have shown their ability to differentiate into a wide variety of cell types, including chondrocytes, osteocytes, and adipocytes in appropriate culture conditions [1]. MSCs can be isolated from a large panel of tissues including bone marrow, adipose tissue, and articular synovial fluid [2]. MSCs mainly exert their regenerative properties through the secretion of bioactive trophic factors that have potent immunomodulatory, proangiogenic, antiapoptotic, antifibrotic, and anti-inflammatory effects [3, 4]. Proinflammatory cytokines (IL-1*β*, TNF-*α*, and interferon-gamma (INF-*γ*)) or interaction with monocytes activates MSCs [5]. In response to those activating stimuli, MSCs exert their therapeutic properties by secreting immunomodulatory factors such as indoleamine 2,3-dioxygenase (IDO), hepatocyte growth factor (HGF), prostaglandin E_2_ (PGE_2_), and transforming growth factor-beta (TGF-*β*) [4, 6–8]. Unfortunately and despite promising results, direct injection of MSCs suffers from certain limitations including massive cell death upon injection [9] and risk of cell leakage outside the site of injection due to the propensity of MSCs to migrate [10, 11].

To overcome both these limitations, several studies have proposed to encapsulate MSCs by using cytoprotective biomaterials [9, 12, 13]. The multipotency of MSCs after encapsulation, for example, has been exploited for the treatment of several pathologies including osteoarticular diseases, diabetes, cancer, cardiovascular pathologies, angiogenic diseases, and skin injury [14, 15]. However, their immunomodulatory properties after encapsulation have been little investigated.

Alginate is nowadays the most largely investigated and characterized hydrogel for the development of cell encapsulation technology [16]. However, alginate particles are sensitive toward nongelling agents such as sodium ions and in physiological solution, a calcium replacement occurs, leading to destabilization and rupture of the gel [17]. Furthermore, the calcium, generally used to cross-link alginate hydrogels, has been shown to exert an immunostimulatory effect *in vitro* and *in vivo* [18]. Our laboratory has developed an injectable and biocompatible cellulose-based hydrogel that can be used for the arthroscopic injection of MSCs in cartilage defects. The Si-HPMC hydrogel supports (i) the three-dimensional culture of MSCs and their controlled differentiation towards a chondrogenic phenotype [19], (ii) the transplantation of MSCs in subcutis of nude mice and the *in vivo* formation of chondrogenic nodules [20], and (iii) the repair of osteochondral lesions in rabbit [20]. Despite this large body of evidence suggesting that Si-HPMC exhibits the required properties, it has never been used to encapsulate MSCs in a particulate form, although it is well known that encapsulation in spherical particles improves injectability, biocompatibility, and stability.

The aims of this work were first to demonstrate the feasibility of generating particles with Si-HPMC hydrogel and to compare their mechanical and physicochemical properties with alginate particles and second to evaluate the *in vitro* viability and bioactivity of MSCs (human adipose-derived MSCs, hASC) encapsulated in Si-HPMC particles.

## 2. Materials and Methods

### 2.1. Materials

Sodium alginate (Protanal™LF10/60FT) and hydroxypropyl methylcellulose (HPMC) (Methocel™E4M) were purchased, respectively, from FMC Biopolymer and Colorcon-Dow chemical (Bougival, France). Glycidoxypropyltrimethoxysilane (GPTMS) was obtained from Acros (Geel, Belgium). Hank's balanced sodium salt (HBSS), Dulbecco's modified eagle medium (DMEM) high glucose (4.5 g/L), phosphate-buffered salt (PBS) without calcium chloride and magnesium chloride, penicillin/streptomycin, and trypsin/EDTA (0.05%/0.53 mM) were obtained from Invitrogen (Paisley, UK). Calcium chloride, 4-(2-hydroxyethyl)-1-piperazineethanesulfonic acid (HEPES), olive oil, fluorescein isothiocyanate- (FITC-) dextrans, collagenase crude type I A, trypan blue, sodium citrate, and trichloroacetic acid (TCA) were purchased from Sigma-Aldrich (St. Louis, MO, USA). Fetal calf serum (FCS) was purchased from Dominique Dutscher (Brumath, France). Live/Dead Viability/Cytotoxicity kit and Quant-iT PicoGreen dsDNA assay kit were, respectively, obtained from Molecular Probes (Leiden, The Netherlands) and Thermo Fisher (Waltham, MA, USA). PGE_2_ EIA Kit-Monoclonal and Human HGF Duo Set ELISA were purchased from Cayman Chemical and R&D Systems, respectively.

### 2.2. Preparation of Particles

Alginate particles were obtained using a dropwise method in CaCl_2_ as previously described [21]. First, sodium alginate was sterilized by steaming (134°C, 4 minutes) and then dissolved in sterile PBS (2% *w*/*v*). Particles were obtained by extruding this solution through a 31G needle into a stirred solution of 100 mM calcium chloride. After 15 minutes, alginate particles were collected by filtration, washed in HEPES buffer, and stored at room temperature. Synthesis of Si-HPMC was performed by grafting 14.24% (*w*/*w*) of GPTMS onto HPMC in heterogeneous medium as previously described [22]. Si-HPMC powder was solubilized (3% *w*/*v*) in 0.2 M NaOH under constant stirring for 48 h. The solution was then sterilized by steaming (121°C, 20 minutes). To initiate the formation of a cross-linked Si-HPMC, the solution was mixed with 0.5 volume of 0.26 M HEPES buffer (pH 3.6). To obtain Si-HPMC particles, an oil dispersion protocol was performed. The solution of Si-HPMC/HEPES was injected into olive oil under stirring. To optimize the encapsulation process, three dispersion parameters were studied: time (1 h or 1 h and 30 min), temperature (room temperature or 37°C), and stirring speed (250 rpm or 400 rpm). Si-HPMC particles were collected by filtration, washed in HEPES buffer, and stored at room temperature.

### 2.3. Particle Characterization

#### 2.3.1. Shape and Size

Particles were observed under light microscopy (Leica confocal system, TCS NT/SP series, Germany) to investigate their shape. A Mastersizer 3000 Laser (Malvern Instruments, UK) was used for particle size and reproducibility analysis.

For the subsequent analyses, alginate and Si-HPMC particles of 1 ± 0.2 mm diameter were selected under light microscopy.

#### 2.3.2. Particle Diffusion Properties

To study the diffusion properties, alginate and Si-HPMC particles were immobilized at the bottom of Lab-Tek chambers and incubated in 1 mg/mL solutions of fluorescently labeled dextran (Mw 20, 250, or 2000 kDa), for 2 h and 30 min at room temperature. At specific time points, particles were assessed by confocal microscopy (Nikon A1R Si, Champigny-sur-Marne, France; excitation 488 nm, emission 520 nm). Image analysis was performed with NIS-Elements software, and maximum fluorescence intensity was determined inside particles, as well as the fluorescence intensity of the FITC-dextran solution (outside particles). A ratio of internal to external fluorescence was then calculated, a ratio of 1 indicating that fluorescence intensity was identical in a particle and the surrounding solution. The results are presented as the ratio internal/external fluorescence as a function of time.

#### 2.3.3. Particle Mechanical Properties

Mechanical properties of alginate and Si-HPMC particles were investigated by subjecting them to a compressive force between 2 parallel plates for 30 s (Microsquisher, CellScale). The force (*μ*N) and the displacement (*μ*m) were measured using a microscale test system equipped with an integrated image analysis module. The results are expressed as the force applied versus the recorded displacement. Young's modulus was calculated according to the manufacturer's recommendations and the standard expression *E* = stress/strain = (*F*/*A*)/(∆*l*/*l*_0_) where *E* is Young's modulus; *F* is the force applied on a particle; *A* is the area through which the force is applied; Δ*l* is the displacement; and *l*_0_ is the initial diameter of the particle.

### 2.4. Isolation and Culture of hASC

Human adipose-derived MSCs (hASC) were isolated from subcutaneous adipose tissue of patients undergoing liposuction after having given their informed consent as described elsewhere [23]. All protocols were approved by the French national ethical committee. Briefly, lipoaspirate was washed five times in HBSS and then digested for one hour at 37°C under constant stirring, in a solution of 0.025% collagenase in HBSS. The collagenase treatment was inactivated by adding an equal volume of DMEM high glucose containing 1% penicillin/streptomycin and 10% FCS (complete medium). After 5 minutes of centrifugation (260*g*, 4°C), the lower phase containing the stromal vascular fraction was collected, homogenized, filtered through a 70 *μ*m cell strainer, and centrifuged for 8 minutes (260*g*, 4°C). Cells were suspended in complete medium, seeded at 5000 cells/cm^2^, and incubated at 37°C in a humidified atmosphere containing 5% CO_2_. After 2-3 days of incubation, the nonadherent cells were removed by successive washes. Before encapsulation, hASC surface marker expression was characterized by flow cytometry (data not shown) as previously described [19].

### 2.5. hASC Encapsulation

Cells were tested at passage 5 (population doubling level (PDL) of 13.6). hASC were harvested using trypsin/EDTA, counted, and then loaded into alginate and Si-HPMC particles. For alginate, 2.10^6^ hASC (100 *μ*L) were suspended in 1 mL of a sterile solution of 2% sodium alginate. After homogenization, the mixture was extruded through a 31G needle into a sterile solution of calcium chloride. The particles were collected by filtration, washed with HEPES buffer, and cultured in complete medium. For Si-HPMC, 2.10^6^ hASC (100 *μ*L) were also mixed with 1 mL of Si-HPMC hydrogel, after induction of cross-linking by adding HEPES buffer as previously described [22]. The microparticles were obtained by introducing the mixture into olive oil under stirring at 250 rpm and at room temperature. After 1 h and 30 min of stirring, the microparticles were collected by filtration, washed with HEPES buffer, and incubated in complete medium.

The average number of encapsulated hASC per particle was assessed, every week, by using twenty alginate and Si-HPMC particles for each measure. Alginate particles were disrupted into a 60 mM sodium citrate for 15 minutes, and Si-HPMC particles were incubated in Tris/EDTA buffer for 30 seconds then disrupted by ultrasonication for 15 seconds (Misonix XL2000 Microson Ultrasonic Cell Disruptor). The average number of encapsulated hASC was estimated by DNA quantification which was performed on cell lysate using a Quant-iT PicoGreen dsDNA assay kit, following the manufacturer's instructions as previously described [24, 25].

### 2.6. hASC Viability and Biofunctionality

Encapsulated cells in alginate and Si-HPMC were cultured and incubated for up to 1 month at 37°C in a humidified atmosphere containing 5% CO_2_. Complete medium was changed every 2 days.

hASC viability after encapsulation in alginate and Si-HPMC particles was followed from 24 h to 1 month of culture using a Live/Dead Viability/Cytotoxicity kit and determination of encapsulated cells. Alginate and Si-HPMC particles were recovered and washed in PBS for 20 seconds. Then, particles were incubated for 45 minutes in the combined Live/Dead assay reagents. Labeled cells were followed by confocal microscopy using an inverted fluorescence microscope (Nikon Eclipse TE 2000 E, Badhoevedorp, The Netherlands). The viability was calculated using the Volocity Software V6.2 (PerkinElmer, MA, USA).

In separate experiments, 1 week after encapsulation in alginate or Si-HPMC, hASC were stimulated for 72 hours with proinflammatory molecules (TNF-*α* + IFN-*γ* at 20 ng/mL) in complete medium, as previously described [26]. For each condition, 20 alginate or Si-HPMC particles were used. In order to quantitatively assess the production of soluble molecules (IDO, PGE_2_, and HGF), commercially available kits were used. IDO enzymatic activity was measured by tryptophan-to-kynurenine conversion with photometric determination of kynurenine concentration in the supernatant. Briefly, 60 *μ*L of cell supernatant (conditioned medium) was transferred to a 96-well culture plate, and 30 *μ*L of 30% trichloroacetic acid solution was added for 30 minutes at 50°C. After centrifugation, 75 *μ*L of samples was added to 75 *μ*L of freshly prepared Ehrlich's solution, and absorbance was read at 450 nm. PGE_2_ and HGF were measured in conditioned medium using ELISA kits according to the manufacturer's recommendations. The secretion of these biofactors in supernatant was normalized to the number of encapsulated hASC in both alginate and Si-HPMC particles.

### 2.7. Statistical Analysis

All experiments were performed with replicate samples from independent conditions (*n* = 3 for particle size, *n* = 3 for diffusion properties, *n* = 4 for mechanical testing, *n* = 3 for cell counts and viability, and *n* = 2 for biofunctionality). The results are presented as the mean of the independent replicates, and the error bars represent the standard error of the mean. The comparative studies of means were performed with GraphPad software by using one-way ANOVA followed by a post hoc test (Fisher's projected least significant difference) with a statistical significance at *p* < 0.05.

## 3. Results

### 3.1. Assessment of the Shape and Size of Particles

**Figure 1:**
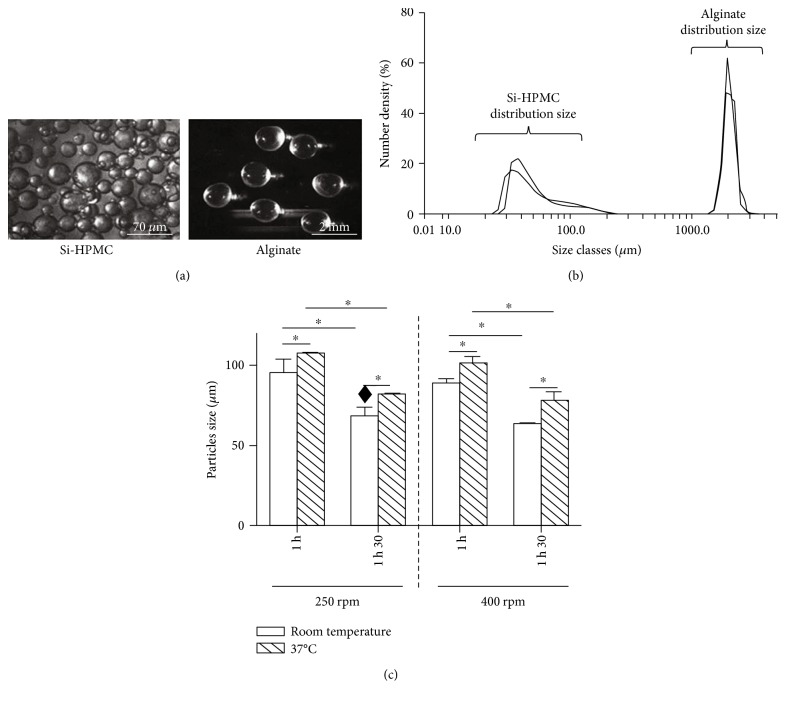
Particle characterization. (a) Representative observation of Si-HPMC and alginate particles obtained by oil dispersion and dripping method, respectively, and observed under light microscope. (b) Dispersity and size distribution of Si-HPMC and alginate particles investigated by laser diffraction technique. (c) Reproducibility and effect of dispersion parameters (time, temperature, and stirring speed) on Si-HPMC particle size. ^♦^The operating conditions selected to generate Si-HPMC particles for the size distribution investigation (Figure 1(b)) and for the following experiments. ^∗^*p* < 0.05. Scale bars = 70 *μ*m and 2 mm for Si-HPMC and alginate particles, respectively.

Si-HPMC and alginate particles were obtained using an oil dispersion and a dripping method, respectively, (Figure 1(a)).

Alginate and Si-HPMC particles possessed a shape uniformity. Prepared with a 31G needle, alginate particles exhibited an average size of nearly 2 mm (1.91 ± 0.2 mm). The laser diffraction size analyzer showed that more than 80% of alginate particles had a size distribution between 1600 and 2100 microns (Figure 1(b)). Moreover, some particles of about 1 mm were also detected. To optimize encapsulation process with Si-HPMC, three dispersion parameters were used: dispersion time (1 h or 1 h and 30 min), temperature (room temperature or 37°C), and stirring speed (250 rpm or 400 rpm).  Figure 1(c) shows the influence of these parameters on Si-HPMC particle size. Increasing temperature from room temperature to 37°C induced a significant increase in particle size from 92 ± 10.1 *μ*m to 108 ± 2 *μ*m at 250 rpm for one hour. This effect of temperature on particle size was observed with all the other conditions. Increasing the dispersion time from 1 h to 1 h and 30 min decreased by approximately 25% the size of particles whatever the stirring speed applied. On the other hand, the variation of stirring speed had no significant effect on the Si-HPMC particle size. For the following experiments, we selected the subsequent dispersion parameters: dispersion time 1 h and 30 min and stirring 250 rpm at room temperature. Under these operating conditions, Si-HPMC particles presented an average size of 68 ± 28 *μ*m with a good reproducibility. Laser analysis showed a size distribution larger for Si-HPMC particles than for alginate ones (Figure 1(b)). We also observed a predominant population from 31 ± 18 to 76 ± 11 *μ*m (69%) and a minor one from 76 ± 11 to 144 ± 23 *μ*m (11%). Furthermore, we detected some larger Si-HPMC particles greater than or equal to 1 mm.

### 3.2. Alginate and Si-HPMC Particle Characterization

**Figure 2:**
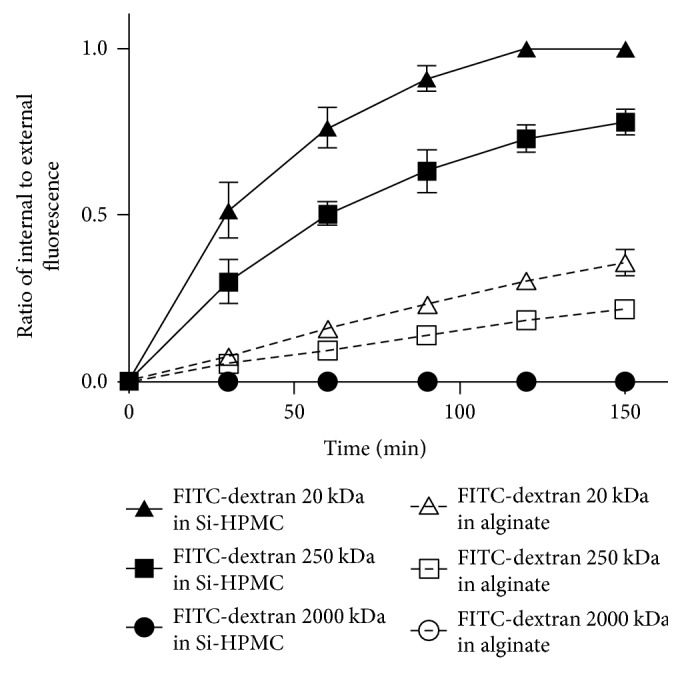
Alginate and Si-HPMC particle diffusion properties. Alginate and Si-HPMC particles were incubated in fluorescently labeled FITC-dextran (Mw 20, 250, and 2000 kDa) solutions for 2 h and 30 min. Fluorescence intensities of particles (inside) and FITC-dextran solutions (outside) were assessed by confocal microscopy. A ratio of internal to external fluorescence was then calculated, and a ratio of 1 indicating that fluorescence intensity was identical in a particle and the surrounding solution. Alginate and Si-HPMC particles of 1 mm diameter were selected for this study. Each test was performed for one particle at a time and repeated at least 3 times.

Diffusion properties of alginate and Si-HPMC hydrogels were investigated after incubation of particles (1 mm in diameter) in 1 mg/mL solutions of FITC-dextran (Mw 20, 250, or 2000 kDa) for 2 h and 30 min. At the beginning of the experiment, particles, observed using confocal microscopy, were not fluorescent at all, and then we noted an increase in the fluorescence intensity in some particles as a function of time. To compare hydrogels, a ratio of internal (i.e., inside a particle) to external (i.e., dextran solution) fluorescence intensity was calculated. For 20 kDa and 250 kDa FITC-dextrans, we noted that both alginate and Si-HPMC particles became fluorescent (Figure 2) and that the intensity ratio increased as a function of time.

On the contrary, when incubated with high molecular weight-labeled dextran (2000 kDa), no fluorescence was detected in particles (circle symbols) and the calculated ratio remained equal to zero. In less than 2 hours, equilibrium of fluorescence intensity (i.e., ratio = 1) was reached for the 20 kDa dextran in Si-HPMC particles (black delta symbol) but not observed for 250 kDa (black square symbol) nor with alginate (white delta and square symbols), indicating that diffusion rate of dextrans was faster in Si-HPMC particles as compared to alginate ones.

**Figure 3:**
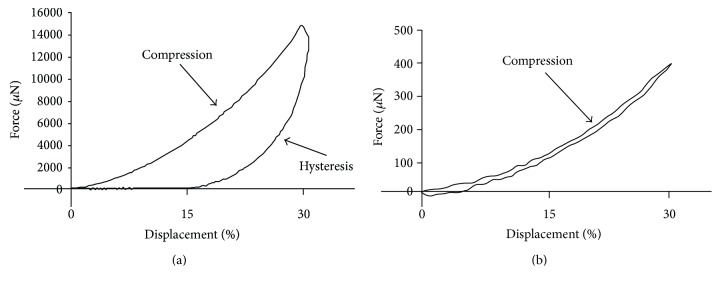
Mechanical properties of the particles. Compressive properties of alginate (a) and Si-HPMC (b) particles were investigated by subjecting them to a 30% compression for 30 seconds. The force (*μ*N) and displacement (%) were recorded, and Young's modulus was determined. Alginate and Si-HPMC particles of 1 mm diameter were selected for this study.

To investigate their mechanical properties, particles of 1 mm size were subjected to a 30% compressive displacement over a period of 30 s (Figure 3).

While an alginate particle required a 14 mN force, a force as low as 0.4 mN was sufficient for Si-HPMC one. This is reflected in the significantly different Young's moduli of 16 ± 0.82 kPa for alginate particles and 0.22 ± 0.06 kPa for Si-HPMC ones. In addition, we noticed that alginate particles had a viscoelastic response as the unloading path was steeper than the loading curve while Si-HPMC particles presented a minimal hysteresis and returned to their original geometry when the compressive force was removed.

### 3.3. Estimation of the Average Number of Encapsulated hASC

**Figure 4:**
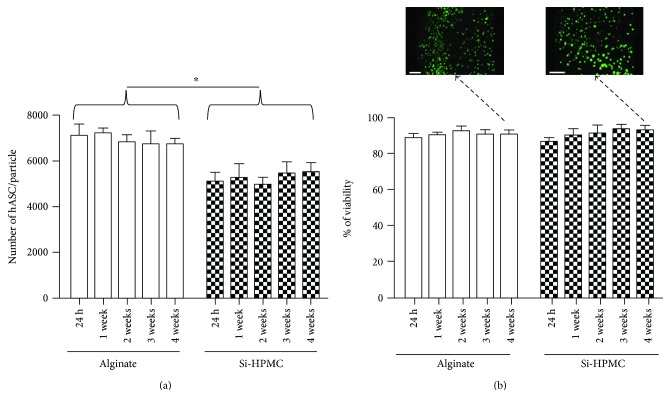
hASC viability after encapsulation in alginate and Si-HPMC. The average number of encapsulated hASC per alginate and Si-HPMC particles was evaluated from 24 h to 1 month. (a) Average number of cells per particle. Alginate particles were dissolved in sodium citrate solution, and Si-HPMC particles were disrupted by ultrasonication. A DNA quantification by PicoGreen has allowed us to estimate the cell number. (b) Cell viability. Using a Live/Dead Viability/Cytotoxicity kit, cells encapsulated in alginate and Si-HPMC particle were assessed from 24 h to 1 month of culture. Viable cells were imaged using a confocal microscope, and viability was calculated with Volocity software. Scale bars = 100 *μ*m. ^∗^*p* < 0.05.

To investigate the average number of encapsulated hASC, alginate and Si-HPMC particles were disrupted by using sodium citrate solution and ultrasonication, respectively. The average number of encapsulated hASC per alginate particle was significantly higher than the one in Si-HPMC particle (Figure 4(a)).

We counted about 7102 ± 514 hASC per alginate particle versus 5100 ± 407 hASC per Si-HPMC particle, 24 hours after encapsulation. It is worth noting that, with regard to a theoretical volume of 3.6 *μ*L (based on a particle mean size), a particle could contain 7200 encapsulated cells, at most. Hence, the encapsulation efficiency, calculated as the ratio of the experimental number of encapsulated cells to the theoretical one, was 97% for alginate and 70% for Si-HPMC.

### 3.4. Evaluation of Encapsulated hASC Viability and Biofunctionality

Whatever the polymer used, the number of encapsulated cells did not significantly change during the 4-week study. For the duration of the study, the viability of encapsulated hASC in Si-HPMC and in alginate was 90.8 ± 3.8% and 90.5 ± 2.4%, respectively (Figure 4(b)). No significant difference between the viability of hASC encapsulated in Si-HPMC and in alginate was observed. The distribution in cell viability along the radial direction of both alginate and Si-HPMC particles was uniform, and no accumulation of dead cells in the center of the particles was detected (Figure 4(b)). Furthermore, viable cells do not seem to physically interact among themselves and formation of cell clusters was not observed. As such, hydrogels may provide optimal conditions for cell survival, since clusters might limit nutrient availability and slow down secretion of biological factors.

To assess the secretion properties of encapsulated hASC, cells were treated for 72 hours with proinflammatory molecules (TNF-*α* + IFN-*γ*) as previously described [26]. In the control condition, when encapsulated hASC in alginate were not stimulated, the basal concentration of L-kynurenine in supernatant was 6.08 ± 1.4 × 10^−5^ *μ*M per encapsulated hASC.

**Figure 5:**
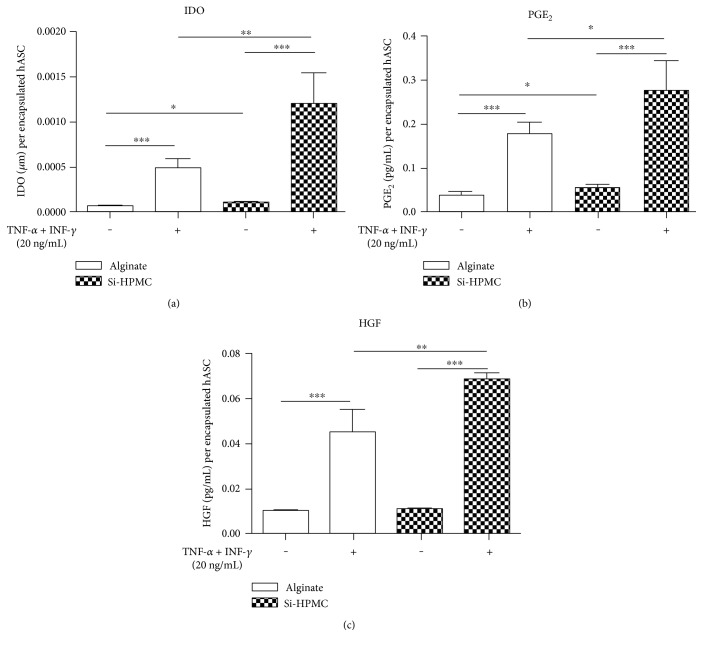
IDO enzyme activity and PGE_2_ and HGF concentrations in supernatants of encapsulated hASC. One week after encapsulation in alginate and Si-HPMC, hASC were stimulated (or not) with TNF-*α* and IFN-*γ* (20 ng/mL each) for 72 h. For each condition, twenty alginate or Si-HPMC particles were then disrupted and encapsulated hASC were counted. IDO enzyme activity (a) was measured by tryptophan-to-kynurenine conversion with photometric determination of kynurenine concentration in the supernatant. PGE_2_ (b) and HGF (c) were measured in cell supernatant using ELISA kits according to the manufacturer's recommendations. The secretion of these biofactors was normalized to the number of encapsulated hASC in both alginate and Si-HPMC particles. Alginate and Si-HPMC particles of 1 mm diameter were selected for this study. ^∗^*p* < 0.05, ^∗∗^*p* < 0.01, and ^∗∗∗^*p* < 0.001.

The stimulation of hASC encapsulated in alginate and Si-HPMC with IFN-*γ* combined with TNF-*α* induced a respective 8-fold and 19.7-fold increase in the concentration of L-kynurenine in supernatant as compared to the control condition (unstimulated encapsulated hASC in alginate) (Figure 5(a)). Moreover, encapsulation of hASC in Si-HPMC showed a concentration of L-kynurenine significantly higher than in alginate.

For PGE_2_, and compared to unstimulated hASC in alginate, we observed a 4.9-fold increase when encapsulated hASC in alginate were stimulated with TNF-*α* + INF-*γ* (Figure 5(b)). The increase was more prominent (7.67-fold increase) when hASC were encapsulated in Si-HPMC. As already observed, encapsulated hASC in alginate or Si-HPMC were able to secrete PGE_2_, and the concentration in supernatant was increased after stimulation with proinflammatory cytokines. hASC encapsulated in Si-HPMC also showed a significantly higher concentration of PGE_2_ in supernatant than hASC encapsulated in alginate.

Compared to unstimulated hASC in alginate, the stimulation of encapsulated hASC in alginate and Si-HPMC with TNF-*α* and IFN-*γ* increased the supernatant concentration of HGF by 4.4 and 6.7, respectively (Figure 5(c)). As previously observed for L-kynurenine and PGE_2_, after stimulation, HGF concentration in supernatant was significantly higher when hASC were encapsulated in Si-HPMC than in alginate.

All these results indicate that hASC encapsulated in alginate or Si-HPMC are sensitive to proinflammatory cytokines and respond to this stimulation by increasing their secretion of IDO, PGE_2_, and HGF. Moreover, higher levels of L-kynurenine, PGE_2_, and HGF were measured in the supernatant of Si-HPMC-encapsulated hASC than those encapsulated in alginate.

## 4. Discussion

MSCs mainly exert their regenerative properties *in vitro* and *in vivo* through the secretion of biofactors [3, 4]. However, the secretion of these therapeutic bioactive factors after encapsulation of MSCs has not yet been proven and investigated for a potential application in inflammatory disease treatment.

In this context, the aims of this study were to evaluate (i) the feasibility of generating particles with Si-HPMC hydrogel and (ii) the *in vitro* viability and bioactivity of hASC encapsulated in Si-HPMC particles. Si-HPMC is a semisynthetic polymer that undergoes condensation and cross-linking when the pH decreases by addition of an acidic HEPES buffer [22]. Unlike alginate which gelation in CaCl_2_ solution is near instantaneous, Si-HPMC cross-linking requires about 40 minutes at room temperature. We verified previously that dropwise method is not appropriate to generate Si-HPMC particles. So we have developed an oil dispersion protocol to obtain Si-HPMC particles. Our results showed that this method is noncytotoxic and appears suitable for hydrophilic materials, as Si-HPMC.

The choice of particle size selection is governed by a compromise between the site of injection, the targeted number of cells to inject, and the desired quality of exchange between particles and their external environment. One of the limiting factors in the use of alginate for encapsulation is that the size of generated particles is dictated by the extrusion needle diameter. In practice, it is difficult to generate particles with needles of a diameter smaller than 31G (availability of needles and high viscosity of polymeric solutions). Extrusion through a 31G needle generated spherical alginate particles with a size of about 2 mm. This size is not compatible with injection in a small animal model like rodents. Theoretically, it would be possible to reduce the size of alginate particles by modifying the extrusion operating conditions (surface tension of alginate solution, concentration of calcium chloride, and curing time particle in gelation bath [27]) or by selecting another method of particle generation like microfluidic technologies. Another option is to select a different polymer to obtain smaller particles. In this study, we generated about 100 *μ*m diameter Si-HPMC particles and we observed that this size was mainly influenced by 2 parameters: temperature and dispersion duration. The following dispersion parameters were chosen: 1 h and 30 min as dispersion time to allow sufficient particle gelation, stirring at 250 rpm to reduce as much as possible shear stress, and room temperature to obtain smaller particles. Under these operating conditions, Si-HPMC particles are smaller than alginate ones as expected but with an important size distribution.

Particle geometry is also a parameter which has an impact on the injectability and biocompatibility of hydrogels. Indeed, it was reported that nonspherical shape with acute angle particles does not promote their injectability (caused by their poor flow rate) and above all induce inflammation by producing important amount of foreign body responses *in vivo* [28, 29]. We observed that both alginate and Si-HPMC particles are spherical making their shape likely appropriate for *in vivo* application.

As the extrusion process of alginate generates particles of more than 1 mm in diameter, we decided, for further experiments, to select both alginate and Si-HPMC particles of about 1 mm size. This allows a consistent comparison of both materials for particle properties and hASC viability and biofunctionality.

This study relies on the diffusion of molecules of interest from outside to inside of the particles (proinflammatory cytokines as stimulants) and inversely (bioactive factors secreted by stimulated MSCs as therapeutic agents). For therapeutic effectiveness, encapsulation should allow a sustained delivery of biofactors and maintain a high local concentration of these molecules in the surrounding tissues. This diffusion is affected by the mechanical stress applied on the particle *in vivo* which strongly depends on elasticity, swelling, and charge density of the hydrogel. These factors and their interactions create a complex environment that determine the diffusion kinetics, rate of bioactive factor release through the particles, and duration [30]. Due to its wide use in diffusion studies as a result of its ease of use [31, 32], we selected FITC-dextran (Mw 20, 250, or 2000 kDa). A particular interest was focused on the 20 kDa FITC-dextran, since proinflammatory cytokines and biofactors secreted by stimulated MSCs have molecular weights ranging from about 10 to about 45 kDa [33–35]. Our results clearly show that Si-HPMC and alginate hydrogels have different diffusion properties. Indeed, FITC-dextrans diffuse faster in Si-HPMC particles than in alginate ones identically sized. This observation could be due to a larger mesh size (i.e., porosity) of Si-HPMC particles compared to alginate ones. This result suggests that MSC encapsulation in Si-HPMC would allow a faster reactivity to proinflammatory cytokines and a faster release of bioactive factors.

Mechanical properties of nonadhesive hydrogels dictate the behavior of encapsulated cells and might impact their viability and bioactivities. For example, high stiffness can decrease the viability of encapsulated cells as described by Wright et al. [36]. Mechanical tests and Young's modulus suggest that alginate particles were stiffer than Si-HPMC ones. Young's modulus value of alginate particles (16 kPa) was similar to previously reported literature values (ranging from 5 to 18 kPa, [37]). After compressive displacement, Si-HPMC particles recover their original shape faster than alginate ones. This difference suggests that Si-HPMC particles would allow a quicker relaxation during and after injection than alginate ones thereby allowing a better behavior of encapsulated cells [38].

Given that MSCs exert their therapeutic potential partly by the secretion of bioactive factors exhibiting immunomodulatory, antifibrotic, proangiogenic, or antiapoptotic properties, encapsulated MSCs must be viable and their average number per particle should be sufficient to obtain an efficient production of these bioactive factors. MSCs were isolated from human adipose tissue which can be obtained easily under local anesthesia. Furthermore, adipose tissue contains MSCs in larger numbers (5% of nucleated cells, [2]). Our results show that the viability of encapsulated hASC in alginate and Si-HPMC particles remained higher than 86% for over a month. The disruption of particles is essential for estimating the average number of encapsulated hASC. Si-HPMC particles cannot be disrupted by enzymatic treatments (data not shown), and only ultrasonication is effective. The average number of hASC encapsulated per alginate particle was higher than in Si-HPMC particle identically sized. This result could be due to a loss of hASC (during their incorporation in precross-linked Si-HPMC hydrogel) or to their leakage from Si-HPMC droplets during the dispersion protocol (1 h and 30 min) since, unlike alginate, Si-HPMC cross-linking is not instantaneous and requires about 40 minutes at room temperature. The number of encapsulated hASC per alginate and Si-HPMC particles remained constant over time, which is consistent with numerous publications reporting a slow cell proliferation rate, often linked to the lack of bioactive molecules in alginate and Si-HPMC hydrogels [39–41]. This long-term *in vitro* viability study confirms that the particle matrix allows the diffusion of nutrients, oxygen, and glucose needed to maintain the viability of encapsulated hASC. Despite the use of large alginate and Si-HPMC particles (1 mm in diameter) for the cell cultures, a good viability of hASC at the center of particles was observed. This strongly suggests that if a suitable diffusion through the center of the particles is obtained, the particle size is not a limiting factor for cell viability. We also noted that the difference of FITC-dextran diffusion between alginate and Si-HPMC particles is not related to a difference in encapsulated hASC viability.

Since they exert anti-inflammatory effects, MSCs seem to be effective in preventing the evolution of various diseases, including Crohn's disease, GVHD (graft-versus-host disease), IBD (inflammatory bowel disease), and osteoarthritis [42–47]. However, there is no study investigating the secretion of therapeutic bioactive factors by encapsulated MSCs. Therefore, we investigated the secretion of 3 soluble factors by hASC after encapsulation. IDO is an enzyme of tryptophan catabolism through kynurenine pathway. IDO activity is directly reflected by the concentration of L-kynurenine in supernatant. The gradual decrease of tryptophan in the environment and kynurenine synthesis leads to the inhibition of T cell and NK cell proliferation and their apoptosis through c-Myc pathway [7]. PGE_2_ inhibits activation, proliferation, and cytotoxicity of immune cells [6, 8, 43]. PGE_2_ has also anti-inflammatory properties by activating the programming of macrophages in anti-inflammatory M2 phenotype which express IL-10 [48]. HGF has also an anti-inflammatory function on dendritic cells [49]. In addition, it is well known that secreted HGF by MSCs has antifibrotic properties [44].

Our results show that encapsulation does not impair the ability of hASC to secrete bioactive trophic factors in a proinflammatory environment. Indeed, after stimulation by IFN-*γ* and TNF-*α*, the concentrations of HGF, PGE_2_, and L-kynurenine in supernatant were found to dramatically increase. Therefore, these results demonstrate that (i) proinflammatory cytokines diffuse within particles to stimulate hASC and (ii) stimulated hASC secrete soluble factors which are released through the particles. Compared to alginate, the lower number of encapsulated hASC in Si-HPMC does not cause any negative impact on their ability to secrete bioactive trophic factors. Moreover, kynurenine, PGE_2_, and HGF concentrations in cell supernatant were always higher when hASC are encapsulated in Si-HPMC particles. This difference confirms that hASC encapsulation in Si-HPMC allows a faster release of bioactive trophic factors in supernatant. This could be due to differences in production of biofactors by encapsulated hASC. These results are promising for the use of encapsulated MSCs in the treatment of inflammatory diseases.

## 5. Conclusion

In this study, we propose a device allowing an extended release of biofactors that can be instrumented for reducing inflammation and fibrosis. MSCs can be encapsulated in spherical Si-HPMC particles and remain viable for a month *in vitro*. Moreover, we demonstrated that encapsulation allows the maintenance of hASC responsiveness to IFN-*γ* and TNF-*α* treatment in terms of IDO, PGE_2_, and HGF secretion. Differences in mechanical and physicochemical properties, including diffusion and reactivity of encapsulated MSCs as compared to alginate ones, bring us to the conclusion that Si-HPMC may be more appropriate for the development of an injectable therapeutic cell encapsulation system for MSC delivery. These results are promising, and further *in vitro* and *in vivo* experiments are under investigation to determine whether encapsulated MSCs may be a relevant strategy to prevent inflammation in degenerative disease such as osteoarthritis.
